# Supplementary material for: Assessing African grey parrots' prosocial tendencies in a token choice paradigm
Source: R Soc Open Sci. 2019 Dec 11;6(12):190696. doi: 10.1098/rsos.190696 (PMC6936274; doi:10.1098/rsos.190696)
Supplement: Supplementary Information [file rsos190696supp1.docx]

| **name** | **time** | **condition** | **colours (selfish/prosocial)** | **choice selfish/prosocial token** |
| --- | --- | --- | --- | --- |
| Bella | before | equal | white/blue | 6/6 |
|  |  | unequal | yellow-green/green | 7/5 |
|  | after | equal | white/blue | 7/5 |
|  |  | unequal | yellow-green/green | 6/6 |
| Jack | before | equal | blue/white | 4/8 |
|  |  | unequal | green/yellow-green | 6/6 |
|  | after | equal | blue/white | 12/0***** |
|  |  | unequal | green/yellow-green | 8/4 |
| Jelo | before | equal | blue/brown | 7/5 |
|  |  | unequal | grey/orange | 4/8 |
|  | after | equal | blue/brown | 3/9 |
|  |  | unequal | grey/orange | 0/12* |
| Kimmi | before | equal | blue/brown | 6/6 |
|  |  | unequal | grey/orange | 6/6 |
|  | after | equal | blue/brown | 5/7 |
|  |  | unequal | grey/orange | 1/11* |
| Lizzy | before | equal | brown/blue | 6/6 |
|  |  | unequal | orange/grey | 4/8 |
|  | after | equal | brown/blue | 7/5 |
|  |  | unequal | orange/grey | 12/0* |
| Nikki | before | equal | blue/white | 6/6 |
|  |  | unequal | green/yellow-green | 5/7 |
|  | after | equal | blue/white | 6/6 |
|  |  | unequal | green/yellow-green | 9/3 |
| Nina | before | equal | brown/blue | 7/5 |
|  |  | unequal | orange/grey | 8/4 |
|  | after | equal | brown/blue | 9/3 |
|  |  | unequal | orange/grey | 12/0* |
| Sensei | before | equal | white/blue | 6/6 |
|  |  | unequal | yellow-green/green | 6/6 |
|  | after | equal | white/blue | 1/11* |
|  |  | unequal | yellow-green/green | 4/8 |

**Supplementary Material**

**Table S1.** Individual characteristics of dyads

| **Dyad** | **Name** | **Sex** | **Clutch** | **Year of birth** |
| --- | --- | --- | --- | --- |
| 1 | Kimmi | F | A | 2014 |
|  | Jelo | F | B | 2014 |
| 2 | Lizzy | F | C | 2014 |
|  | Nina | F | C | 2014 |
| 3 | Bella | F | C | 2014 |
|  | Sensei | M | A | 2014 |
| 4 | Nikki | F | B | 2014 |
|  | Jack | M | B | 2014 |

**Table S2.** Individual preferences for tokens used in equal and unequal condition, assessed before and after testing

*Choices deviate from chance level (0.5) as assessed with a binomial test

**Table S3.** Individual choices of prosocial token across conditions (separately for equal and unequal conditions)

|  |  |  | **mean prosocial choices** | |
| --- | --- | --- | --- | --- |
| **dyad** | **actor** | **condition** | **equal** | **unequal** |
| **1** | Jelo | UNI | 39/60* | 60/60* |
|  |  | ALT | 56/60* | 60/60* |
|  |  | YC | 30/30* | 30/30* |
|  |  | ACC | 56/60* | 29/30* |
|  |  | INACC | 60/60* | --- |
|  |  | SFC | 60/60* | --- |
|  | Kimmi | UNI | 36/60 | 54/60* |
|  |  | ALT | 47/60* | 46/60* |
|  |  | YC | 16/30 | 24/30* |
|  |  | ACC | 31/60 | 29/30* |
|  |  | INACC | 33/60 | --- |
|  |  | SFC | 49/60* | --- |
| **2** | Lizzy | UNI | 8/60* | 0/60* |
|  |  | ALT | 19/60* | 4/60* |
|  |  | YC | 19/30 | 1/30* |
|  |  | ACC | 3/60* | 0/30* |
|  |  | INACC | 16/60* | --- |
|  |  | SFC | 0/60* | --- |
|  | Nina | UNI | 21/60* | 0/60* |
|  |  | ALT | 11/60* | 5/60* |
|  |  | YC | 5/30* | 4/30* |
|  |  | ACC | 14/60* | 0/30* |
|  |  | INACC | 25/60 | --- |
|  |  | SFC | 22/60 | --- |
| **3** | Bella | UNI | 55/60* | 37/60 |
|  |  | ALT | 55/60* | 49/60* |
|  |  | YC | 22/30* | 22/30* |
|  |  | ACC | 52/60* | 20/30 |
|  |  | INACC | 44/60* | --- |
|  |  | SFC | 54/60* | --- |
|  | Sensei | UNI | 55/60* | 38/60 |
|  |  | ALT | 59/60* | 58/60* |
|  |  | YC | 30/30* | 28/30* |
|  |  | ACC | 59/60* | 24/30* |
|  |  | INACC | 52/60* | --- |
|  |  | SFC | 5/60* | --- |
| **4** | Jack | UNI | 12/60* | 41/60* |
|  |  | ALT | 44/60* | 41/60* |
|  |  | YC | 29/30* | 10/30 |
|  |  | ACC | 6/60* | 16/30 |
|  |  | INACC | 37/60 | --- |
|  |  | SFC | 6/60* | --- |
|  | Nikki | UNI | 8/60* | 42/60* |
|  |  | ALT | 44/60* | 32/60 |
|  |  | YC | 20/30 | 13/30 |
|  |  | ACC | 39/60* | 14/30 |
|  |  | INACC | 42/60* | --- |
|  |  | SFC | 39/60* | --- |

*choice deviates significantly from chance level (0.5) as assessed with a two-sided binomial test

The eight birds tested in the current study exhibited great individual differences in token choices across conditions. We will discuss the individual performances on a dyadic level:

1. Jelo and Kimmi differed greatly in their prosocial choices. Jelo consistently chose the prosocial tokens across all conditions, while Kimmi was more selective. Despite exhibiting no prosocial preferences in the UNI, Kimmi switched to a prosocial preference in the ALT and SFC condition. In the other controls, she chose tokens at random level. This indicates that Kimmi did not copy the prosocial preference from Jelo but rather only switched to a prosocial preference when choices could be reciprocated. Interestingly, Kimmi did not develop a preference for the prosocial token in the ACC condition, when she could have maximized her payoff. When the reward distribution was unequal, both birds developed a preference for the prosocial token in all conditions.
2. Lizzy and Nina were the only two birds which showed a preference for the selfish token across conditions and reward distributions (equal and unequal). We can rule out that both birds had a preference for the selfish token at the beginning of the study; consequently, either both of them have had inherent selfish tendencies or they were copying each other’s preferences.
3. Bella and Sensei both exhibited a preference for the prosocial token in almost all conditions. Interestingly, Sensei reversed this preference only in the SFC, where he showed a strong preference for the selfish token. With an unequal reward distribution, both birds chose at random during the UNI condition but reversed back to a prosocial preference during the ALT, and YC.
4. Jack and Nikki showed the most variability in their preferences, as they both switched from a selfish preference in the UNI to a prosocial preference in the ALT condition. Jack developed a preference for the selfish token in the ACC and SFC, while Nikki exhibited a prosocial preference during the control conditions. In the unequal conditions, both birds exhibited a prosocial preference during the UNI, but only Jack remained with this preference during the ALT while Nikki chose at random. This dyad shows how preferences for certain types of tokens can change throughout the study and increase when choices could be reciprocated as in the ALT. Once acquired, some birds stuck with a preference for either token in the following conditions.

Note that 12 out of 72 sessions were invalid across all dyads in total due to different causes, i.e. lack of motivation or disturbances, and needed to be repeated. Eight sessions were repeated in the alternating condition (for the same dyad Nikki-Jack), and one session each of Inaccessible-Equal, Accessible-Equal, Social Facilitation, and Accessible-Unequal across two different dyads (Jelo-Kimmi & Nikki & Jack). The lack of motivation in the dyad Nikki-Jack was mostly due to Nikki who was gaining weight and not being food motivated anymore preferring to play with the token rather than handing it back for food. After 4 days of pause in the testing in the alternating condition for this dyad and adjusted amount of daily food, Nikki reduced her weight and was motivated again. Two more adjustments of Nikki’s daily food amount in the course of the study were needed to keep her motivated. Two sessions were invalid because of disturbances caused by maintenance work outside the laboratory.

**Table S4.** Contingency tables for each dyad in equal and unequal ALT condition

| **Dyad** | **Condition** | **Actor’s choice trial *n*** | **Partner’s choice trial *n*-1** | |  |
| --- | --- | --- | --- | --- | --- |
|  |  |  | *Selfish* | *Prosocial* | *Χ^2^* Test |
| 1 | equal | *Selfish* | 0 | 13 | *Χ^2^ =* 1.19, p = 0.276 |
|  |  | *Prosocial* | 4 | 43 |  |
|  | unequal | *Selfish* | 0 | 11 | NA |
|  |  | *Prosocial* | 0 | 49 |  |
| 2 | equal | *Selfish* | 32 | 9 | *Χ^2^ =* 1.13, p = 0.287 |
|  |  | *Prosocial* | 17 | 2 |  |
|  | unequal | *Selfish* | 52 | 4 | *Χ^2^ =* 1.56, p = 0.212 |
|  |  | *Prosocial* | 3 | 1 |  |
| 3 | equal | *Selfish* | 0 | 5 | *Χ^2^ = 0.09*, p = 0.761 |
|  |  | *Prosocial* | 1 | 54 |  |
|  | unequal | *Selfish* | 0 | 11 | *Χ^2^ = 0.46*, p = 0.496 |
|  |  | *Prosocial* | 2 | 47 |  |
| 4 | equal | *Selfish* | 5 | 13 | *Χ^2^ = 0.11*, p = 0.745 |
|  |  | *Prosocial* | 10 | 32 |  |
|  | unequal | *Selfish* | 7 | 15 | *Χ^2^ = 0.97*, p = 0.325 |
|  |  | *Prosocial* | 17 | 21 |  |

**Table S5.** Overview and the testing order of conditions given as an example for the dyad Nikki (Bird A) and Jack (Bird B)

|  |  | Bird's role | |  | |  | |  |
| --- | --- | --- | --- | --- | --- | --- | --- | --- |
| Test | reward distribution | Actor | Partner | | Type of test | | Nb of session x trial | |
| Token choice preference | equal | Bird A |  | | Training | | 1x12 | |
| Token choice preference | equal | Bird B |  | | Training | | 1x12 | |
| Token association training for the 1st actor | equal | Bird A | Bird B | | Training | | 2x30 | |
| Unilateral condition for the 1st actor | equal | Bird A | Bird B | | Test | | 2x30 | |
| Token association training for the 2nd actor | equal | Bird B | Bird A | | Training | | 2x30 | |
| Unilateral condition for the 2nd actor | equal | Bird B | Bird A | | Test | | 2x30 | |
| Alternating condition | equal | Bird A starts/Bird B | Bird B/ Bird A | | Test | | 2x30 | |
| Alternating condition | equal | Bird B starts/ Bird A | Bird A/ Bird B | | Test | | 2x30 | |
| Yoked control | equal | Bird A starts/Bird B | Bird B/ Bird A | | Control | | 2x30 | |
| Yoked control | equal | Bird B starts/ Bird A | Bird A/ Bird B | | Control | | 2x30 | |
| Familiarisation 2nd table "accessible control" | equal | Bird A |  | | Training | | 1x12 | |
| Familiarisation 2nd table "accessible control" | equal | Bird B |  | | Training | | 1x12 | |
| Absent-partner_Accessible | equal | Bird A |  | | Control | | 2x30 | |
| Absent-partner_Accessible | equal | Bird B |  | | Control | | 2x30 | |
| Absent-partner_Inaccessible | equal | Bird A |  | | Control | | 2x30 | |
| Absent-partner_Inaccessible | equal | Bird B |  | | Control | | 2x30 | |
| Social facilitation | equal | Bird A | Bird B | | Control | | 2x30 | |
| Social facilitation | equal | Bird B | Bird A | | Control | | 2x30 | |
| Food preference | unequal | Bird A |  | | Training | | 1x12 | |
| Food preference | unequal | Bird B |  | | Training | | 1x12 | |
| Token association training for the 1st actor | unequal | Bird A | Bird B | | Training | | 2x30 | |
| Unilateral condition for the 1st actor | unequal | Bird A | Bird B | | Test | | 2x30 | |
| Token association training for the 2nd actor | unequal | Bird B | Bird A | | Training | | 2x30 | |
| Unilateral condition for the 2nd actor | unequal | Bird B | Bird A | | Test | | 2x30 | |
| Alternating condition | unequal | Bird A starts/Bird B | Bird B/ Bird A | | Test | | 2x30 | |
| Alternating condition | unequal | Bird B starts/ Bird A | Bird A/ Bird B | | Test | | 2x30 | |
| Yoked control | unequal | Bird A starts/Bird B | Bird B/ Bird A | | Control | | 2x30 | |
| Yoked control | unequal | Bird B starts/ Bird A | Bird A/ Bird B | | Control | | 2x30 | |

**Figure S1.** Prosocial choices across test conditions per dyad

**Video 1.** The video shows the test procedure for all test and control conditions.
